# AISIM: evaluating impacts of user interface elements of an AI assisting tool

**DOI:** 10.1371/journal.pone.0322854

**Published:** 2025-05-22

**Authors:** Kannika Wiratchawa, Yupaporn Wanna, Prem Junsawang, Attapol Titapun, Anchalee Techasen, Arunnit Boonrod, Vallop Laopaiboon, Nittaya Chamadol, Sahan Bulathwela, Thanapong Intharah

**Affiliations:** 1 Visual Intelligence Laboratory, Department of Statistics, Faculty of Science, Khon Kaen University, Khon Kaen, Thailand; 2 Department of Surgery, Faculty of Medicine, Khon Kaen University, Khon Kaen, Thailand; 3 Faculty of Associated Medical Sciences, Khon Kaen University, Khon Kaen, Thailand; 4 Department of Radiology, Faculty of Medicine, Khon Kaen University, Khon Kaen, Thailand; 5 Cholangiocarcinoma Research Institute, Khon Kaen University, Khon Kaen, Thailand; 6 Centre for Artificial Intelligence, University College London, London, United Kingdom; Simon Diedong Dombo University of Business and Integrated Development Studies, GHANA

## Abstract

While Artificial Intelligence (AI) has demonstrated human-level capabilities in many prediction tasks, collaboration between humans and machines is crucial in mission-critical applications, especially in the healthcare sector. An important factor that enables successful human-AI collaboration is the user interface (UI). This paper evaluated the UI of BiTNet, an intelligent assisting tool for human biliary tract diagnosis via ultrasound images. We evaluated the UI of the assisting tool with 11 healthcare professionals through two main research questions: 1) did the assisting tool help improve the diagnosis performance of the healthcare professionals who use the tool? and 2) how did different UI elements of the assisting tool influence the users’ decisions? To analyze the impacts of different UI elements without multiple rounds of experiments, we propose the novel AISIM strategy. We demonstrated that our proposed strategy, AISIM, can be used to analyze the influence of different elements in the user interface in one go. Our main findings show that the assisting tool improved the diagnostic performance of healthcare professionals from different levels of experience (OR  = 3.326, *p*-value <10^−15^). In addition, high AI prediction confidence and correct AI attention area provided higher than twice the odds that the users would follow the AI suggestion. Finally, the interview results agreed with the experimental result that BiTNet boosted the users’ confidence when they were assigned to diagnose abnormality in the biliary tract from the ultrasound images.

## Introduction

Artificial Intelligence (AI) and Machine Learning (ML) are well known for performing specialized tasks extremely well, *e.g.* playing games, identifying objects, and performing diagnosis tasks, compared to humans. However, when these models made mistakes, they failed terribly. AI and ML models also tend to make naïve mistakes that humans would rarely fall prey to, such as adversarial examples (intentionally fooling AI by modifying a few pixels) or noise (unintentionally modifying the input, which causes the AI to make a mistake) [1–4]. Such errors are unacceptable in healthcare tasks.

Recent studies have highlighted critical ethical considerations when deploying AI-assisted tools in healthcare settings. Bonagiri *et al.* [5] and Katirai [6] emphasized that while AI can enhance diagnostic capabilities, it raises concerns about patient privacy, data security, and algorithmic bias that could disproportionately affect certain demographic groups. A comprehensive review by Islam [7] found that healthcare AI systems often reflect historical biases in medical data, potentially perpetuating healthcare disparities if not carefully monitored and adjusted.

Public trust and acceptance of AI in healthcare remains a complex challenge. Survey research by Arigbabu *et al.* [8] and Kutsenko *et al.* [9] revealed that while patients generally support AI augmenting medical decision-making, they express concerns about transparency and accountability in how AI suggestions influence their care. Nuthakki [10] documented that patients strongly prefer being informed when AI tools are used in their diagnosis, highlighting the importance of maintaining clear communication in the patient-doctor relationship. Furthermore, recent work by Maleki and Forouzanfar [11] and Ciecierski-Holmes *et al.* [12] demonstrated that AI model performance can vary significantly across different healthcare settings and patient populations, emphasizing the need for continuous evaluation and careful integration of AI tools into clinical workflows.

Therefore, it is important to develop AI systems that can collaborate with or assist humans in their tasks. The UI that links the AI model to the user plays the critical role of facilitating the collaboration between the user and the machine. In this work, we analyze a user interface, which was developed along with an AI model that aims to classify input ultrasound images of the human biliary tract as normal or as being one of 14 biliary tract abnormalities.

The need for AI to assist in diagnosing the human biliary tract through ultrasound images is vivid for two reasons. Firstly, ultrasonography is one of the most versatile tools which allow healthcare practitioners to investigate internal organs. It is highly accessible and easier to operate compared to Computerized Tomography (CT), MRI (Magnetic Resonance Imaging), and even X-ray [13,14]. Secondly, diagnosis via ultrasound images is, nevertheless, extremely hard due to the device being operator-dependent and needing years of experience before being able to interpret the echo patterns.

In this case, we analyze BiTNet [15]. One of BiTNet’s applications is the assisting tool in which healthcare practitioners upload ultrasound images of the human biliary tract to the system for suggestions from the AI. BiTNet has demonstrated that it improved users’ diagnosis performance on the human biliary tract, ranging from general practitioners (GP) to expert hepatobiliary radiologists.

The contribution of this work is multifold. To the best of our knowledge, this is the first work that evaluated a UI of an intelligent assisting tool for human biliary tract analysis via ultrasound image. This first contribution focuses on studying the influence of the assisting tool as a whole that whether the assisting tool improves users’ performance and influences users’ decision. For the second contribution, this work further analyses how different user interface elements within the assisting tool influence users’ decisions, which is a key missing contribution in the BiTNet manuscript. To analyze the user interface elements, we further propose AISIM, which measures the similarity between the human decision and the AI suggestion to understand the influence of the assisting tool and the effect of different user interface elements through rigorous statistical methods, mixed and fixed effect multivariate logistic regression models.

The paper is organized by first reviewing work related to intelligent assisting tools, user interface analysis in various healthcare applications, and briefly describe BiTNet and its user interface elements which is the main instrument that we focus. Then the methodology section explains four main aspects of our research: AISIM, research question and analysis, user experiment, and user interview questions. Next, the result section discusses the experimental results and the user interview results. Finally, we summarize the findings from our user experiments in the conclusion section.

## Related work

In this section, we review prominent works related to AI assisting tools and the evaluation of user interfaces in assisting tools. All tools discussed in this section were developed as AI and human symbiosis systems to help in healthcare tasks. The last sub-section is dedicated to reviewing BiTNet [15], in which resides the assisting tool we focus our study of this paper.

### AI assisting tools

In this section, we survey AI assisting tools proposed to support healthcare practices and similar mission-critical settings. First, we review AI tools that were evaluated only on the AI model without testing their usability. Gibson *et al.* [16] developed the Dense Dilated Convolutional Network that segments abdominal organs in CT images to guide radiologists when diagnosing the CT image. Bar-Shira *et al.* [17] proposed a super-resolution model to enhance mammogram images that help radiologists in breast lesion localization tasks. Furthermore, Bano *et al.* [18] developed AutoFB to help sonographers estimate fetal biometry, such as fetal size and weight, from fetal ultrasound images via image segmentation models, i.e., the U-Net and Deeplabv3 model.

On the other hand, many works evaluated the AI assisting tools on their ability to improve user performance. Cai *et al.* [19] proposed Hello AI, an AI assisting tool for the prostate cancer segmentation and grading task of pathologists via whole slide imaging (WSI). In this work, they interviewed the participants to identify additional needs to add to the tool after the user testing phase of the vanilla user interface. Additionally, Cai *et al.* [20] proposed SMILY for prostate cancer diagnosis through WSI images. The task of the tool is to retrieve and show similar WSI images from diagnosed patients to the pathologist for reference when diagnosing the new WSI image. Schaekermann *et al.* [21] developed an ambiguity-aware AI assistant that showed an expert’s explanation in the user interface when a sleep technologist’s diagnosis of biosignal data conflicted with the AI prediction. Xie *et al.* [22] proposed CheXplain, an assisting tool that supports physicians in chest X-ray image analysis tasks. The tool was designed through a paired survey study, a user-centered design for the low-fidelity prototype, and an integrated high-fidelity prototype. Calisto *et al.* [23] developed the BreastScreening-AI tool to support radiologists in diagnosing multimodality breast cancer by showing cancer detection results.

Beyond healthcare, similar undertakings are also evident in other fields such as AI-assisted education. In a recent work by Bulathwela *et al.* [24], content-flow bar, an intelligent UI component that summarises educational concepts embedded in videos to accelerate relevance judgments, uses statistical testing with user interaction logs, fused with a user study to determine the usability of the AI component. A paired non-parametric hypothesis test (Mann–Whitney U test) with activity signals such as clicks and watch time is analyzed for the control and intervened conditions followed by a questionnaire [25]. This approach is similar to this work that uses statistical modeling and user feedback to analyze the utility of the UI components.

Furthermore, Intharah *et al.* proposed BiTNet [15], an assisting tool that aims to work directly with practitioners ranging from GPs to radiologists to help them diagnose abnormalities in the human biliary tract via ultrasound images. Although the paper evaluated both AI model accuracy and users’ performance improvement when using the tool, they did not analyze which element in the user interface affects the user’s decisions. In our work, we analyze BiTNet further to study the impacts of different user interface elements of the AI system that cause the users’ performance improvement.

### The evaluation of user interfaces in assisting tools

To evaluate the impacts of assisting tools on the users, in [25–29] opinions of the participants were directly extracted through interview questions. On the other hand, [20,30,31] tested their hypothesis with the mixed-effect statistics. Calisto [23] and Intharah [15] tested the hypotheses with ANOVA and t-test, respectively.

In addition, Schaekermann *et al.* [21] compared the performance of the user interface via statistical testing and user interview questions. In this work, we introduce AISIM to evaluate the impact of different user interface elements via a mixed-effect model and extract opinions through the interview sessions. Additionally, in our interview session, we applied Thematic analysis [32], a qualitative research method used to identify, analyze, and report patterns (themes) within data, providing a rich and detailed understanding of the dataset. Thematic analysis is recognized for its flexibility and adaptability to various research frameworks, making it suitable for diverse studies. It offers clear guidelines and enables a nuanced interpretation of the open-ended interview.

### BiTNet

**Fig 1:**
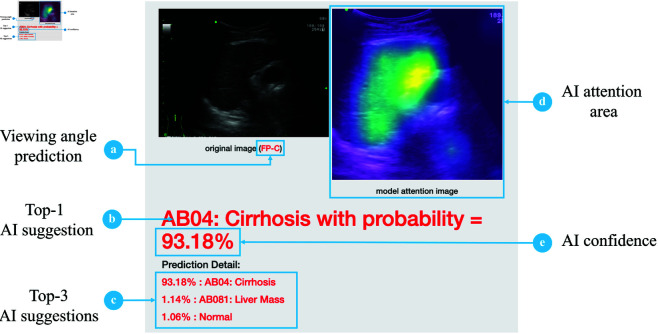
Explaining the user interface elements of the intelligence assisting tool.

**Fig 2:**
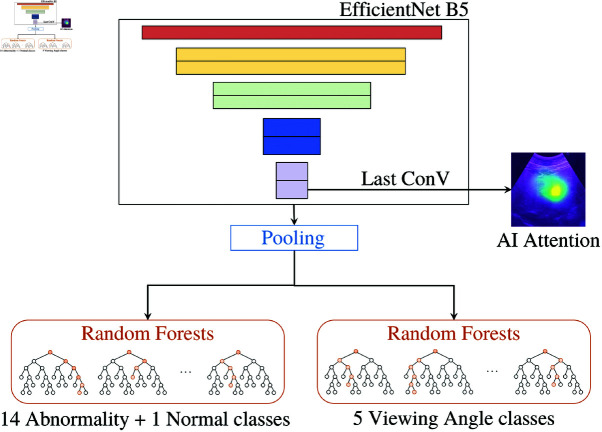
Architecture of the BiTNet model.

In this work, our studies revolve around BiTNet assisting tool [15] as our primary focus tool. It was proposed in 2023 as a web application where healthcare practitioners can upload an ultrasound image of the human biliary tract to obtain an AI suggestion. Its main task is to classify the input image as one of 14 abnormalities presented in a human biliary tract, such as Fatty Liver, Bile Duct Dilatation, and Liver Mass. Besides, it can distinguish a normal image from abnormal classes, a 15-class classification. Along with the main prediction (b: AI suggestion), the user interface presents the model prediction confidence (e: AI confidence), a model attention image (d: AI attention area), the viewing angle prediction of the input ultrasound image (a: viewing angle prediction), and the top-3 most likely prediction (c: Top-3 AI suggestions), illustrated in Figure 1. The model behind BiTNet, see Figure 2, is a hybrid model with EfficientNetB5 [33] as a backbone model. BitNet has two Random Forest Classifiers [34] attached to the last pooling layer of the EfficientNet model to solve the overconfidence issue, which biases the user’s decision when working with an AI model. The model takes an ultrasound image as the input. One Random Forest is trained to predict 15 abnormality classes (14 abnormalities + 1 normal), and another Random Forest is trained to predict 5 classes of viewing angles. Furthermore, the last convolutional layer of the EfficientNet is modified with GradCam [35] to show the model attention area.

BiTNet as a model, achieved 82% AUC on 15-class classification and as an assisting tool showed to improve diagnostic accuracy of all four groups of doctors: GPs, Resident Radiologists, Non-hepatobiliary Radiologists (Non-HB), and Hepatobiliary Radiologists (HB), by 18%.

In this work, we used the user experiment results from BiTNet and analyzed them further to find out about the impacts of BiTNet’s different user interface elements. Due to the experiments in BiTNet testing all the UI elements simultaneously, in our work, we propose a technique to analyze each different user interface element from the results with the AISIM strategy.

## Statistical methods in the study

### Mixed-effects logistic regression for assisting tool evaluation

Mixed-effects logistic regression [36] is a robust statistical method for analyzing hierarchical or nested data, particularly in the case of binary outcome variables. This approach enables the simultaneous evaluation of multiple predictors while accounting for variability within groups by incorporating random effects. It is especially useful when observations are nested or grouped, such as in repeated measurements taken from the same individual. The model accommodates fixed effects (e.g., socioeconomic status or education levels), which remain constant across individuals, and random effects, which capture variability within groups or clusters (e.g., speakers or subjects). The random effects represent hierarchical structures, allowing each group (e.g., individual participants) to have its own intercept or slope, thereby adjusting for unobserved within-group variability. A key application of logistic mixed-effects models is in analyzing binary outcome variables. In these cases, the log odds of the outcome are modeled as a linear combination of risk factors, incorporating both fixed and random effects to provide a more comprehensive understanding of the data structure.

In our study, to answer the first research question, we assessed the influence of an assisting tool on participants’ diagnostic decisions, with a focus on diagnostic performance, particularly diagnostic accuracy, and similarity. Additionally, we examined the effect of radiologist experience levels on these outcomes. Since diagnostic accuracy and similarity were measured as binary repeated measures outcomes, diagnostic status data were collected twice from the same participants, with each radiologist interpreting multiple images. This created a hierarchical data structure with interrelated repeated measurements, and we included both fixed effects (assistance status, radiologist experience) and random effects (radiologists, images) to assess the impact of the AI assisting tool. We applied a logistic mixed-effects model (LMM), incorporating assistance status and radiologist experience as fixed effects while treating radiologists and images as random effects to account for within-subject and image-based variability. To evaluate the effect of AI assistance, we conducted a likelihood ratio Chi-square test, comparing a full model that included AI assistance with a reduced model excluding this variable. This approach allowed us to assess the statistical significance of AI assistance on participants’ diagnostic decisions.

Furthermore, we assessed the impact of the assisting tool on radiologists’ diagnostic similarity in cases where the AI provided an incorrect diagnosis. The mixed-effects model was applied to two subsets of cases: (1) where the top-3 AI predictions were incorrect, and (2) where only the top AI prediction was incorrect. For both subsets, we used a likelihood ratio Chi-square test to compare the full model with a reduced model that excluded the fixed effect of the AI assistance variable. This analysis provided insights into how the accuracy of AI suggestions influenced radiologists’ decisions while also accounting for the effect of radiologist experience levels.

### Multivariate binary logistic regression for UI elements evaluation

Multivariate binary logistic regression is a statistical approach to assess the relationships or influence between a dichotomous outcome (dependent variable) and multiple independent variables. It calculates the probability of an event occurring based on multiple independent variables, making it particularly useful for analyzing repeated measures or nested/clustered data. This technique is often applied when outcomes are measured repeatedly for the same individual or in clustered data structures where multiple individuals belong to a group or cluster [37,38].

In this study, we are interested in assessing the simultaneous effects of several predictor factors, user interface (UI) elements, on the diagnostic similarities between participants’ decisions and AI suggestions. The predictor variables included AI’s confidence, AI’s attention area, and AI’s viewing angle. To ensure an appropriate experimental design, we categorized the predictor variables into distinct categorical levels to avoid the need for participants to make repeated measures diagnostics based on three UI elements conditions (3!). Each participant diagnosed a set of images under assisted and unassisted conditions. The data from the assisted condition were then grouped into low and high confidences for the AI’s confidence; incorrect, undecided, and correct for the AI’s attention area; incorrect and correct for the AI’s viewing angle. The multivariate binary logistic regression model was employed to evaluate the combined effect of AI’s confidence, attention area, and viewing angle on diagnostic similarity, with observed outcomes derived from repeated measures for the same subjects. An odds ratio model was used to assess the impact of each UI element, allowing us to evaluate their association with the dependent variable while controlling for other covariates. This approach enabled us to determine the statistical significance of each UI element on participants’ diagnostic decisions [39]. It allowed us to assess the statistical significance of UI elements on the influence of radiologists’ diagnostics without multiple rounds of experiments which inhibits expert participants to join the study.

### Impact of the sample size on the effect size

In this study, we calculated the effect size using a logistic mixed-effects model, particularly when comparing full and reduced models. We employed G*Power software [40] with a likelihood ratio Chi-square test to determine the difference in Chi-squares between the models. This test indicates the contribution of the excluded variable to explain the variability in the outcome [36]. We calculated the effect size by G*Power based on Cohen’s ${\text{f}}^{2}$ [41] criterion, with a total sample size set at 11 subjects. We obtained an effect size of *f*^2^ = 0.844 with a significance level of α=0.05 and a test power of 1−β=0.80, which indicates a strong and substantial impact of the independent variables in the model. Despite a relatively small sample size, this effect size suggests that the model can still detect the significant relationships between the assisting tool and outcome variables.

While larger sample sizes are generally preferred for precision [42,43], using 11 subjects with repeated measures provides sufficient power to draw valid conclusions in this study. The mixed-effects model accounts for variability between subjects, allowing for a robust evaluation of fixed effects like UI elements. Repeated measures help increase the total number of data points [36], and with an effect size *f*^2^ = 0.844, this sample size is both interpretable and sufficiently powered for the study’s goals.

## Methodology

### AISIM

In this research, we proposed the AI SIMilarity (AISIM) strategy, which used the similarity between participants’ answers and AI suggestions as a variable in the statistical methods to test hypotheses. The statistical method we proposed in this work is mixed effect logistic regression. We compared two rounds of participants’ answers, assisted (by the AI) and unassisted, while accounting for other effects, such as user interface elements, that might impact the answers. The method comprises fixed and random effects, where fixed effects represent a systematic variation effect that impacts the dependent variable, while random effects represent individual effects. the similarity between AI’s answers and the participants’ answers is defined by the Jaccard similarity between participants’ answers and AI suggestions,


$$Jaccard(UD,AI)=\frac{\left|AI\cap UD\right|}{\left|AI\cup UD\right|}$$

Where *AI* is the AI suggestion for each question, and *UD* is the user decision. hence, |AI∩UD| represents the number of similar answers between AI suggestions and user decisions, and |AI∪UD| is the number of questions.

In this work, we use the proposed AISIM strategy along with the mixed effect statistics to measure the impacts of different UI elements on user decisions without having to test each element individually. To achieve that, the similarity, which we will refer to as AISIM, is set as the dependent variable of the mixed effect statistics.

### Research questions and hypotheses

#### [Q1] Did the assisting tool help improve the diagnosis performance of the users?

##### [H1a] The participants have higher accuracy when assisted with the assisting tool than when unassisted.

To evaluate the hypothesis, we used the mixed-effect logistic regression model to compare the effect of the assisting tool on the accuracy, setting the accuracy as the dependent variable. The outcome variable used in this study is diagnostic accuracy. The accuracy variable takes a value of 1 if the participants’ answers are correct and 0 if they are incorrect. We considered the effects of diagnostic status. The effects variables considered are assistance status (Assistance: takes the value of 1 if assisted and 0 if unassisted) and levels of experience (Experience :0 if general practitioners, 1 if residence radiologist, 2 if non-hepatobiliary radiologists, and 3 if hepatobiliary radiologists). A two-level mixed-effects logistic regression model (Models 1–2) [44] was employed to investigate the effect of the AI assisting tool on diagnostic status. In these models, Assistance was considered as a fixed effect, while individual participants (*i*), images (*j*), and Experience (*k*) were treated as random effects. The models assumed that the observations ($y_{111},\ldots ,y_{ijk}$) were correct or incorrect, independent of the diagnostic outcome. The response variable *y*_*ijk*_ was modeled as a Bernoulli distribution, $Y_{ijk}∼\text{Bernoulli}\;(\pi_{ijk})$, where $\pi_{ijk}$ represents the probabilities of obtaining a correct diagnostic outcome for each participant (*i*), image (*j*), and Experience (*k*). Accordingly, based on the dependent variable as a diagnostic status, our mixed-effects logistic regression model to be fitted can be written as:


$$\text{Logit}(\pi |{\text{Asst}}_{i},{\text{Exp}}_{k},U_{k},W_{jk})=\beta_{0}+\beta_{1}{\text{Asst}}_{ijk}+\beta_{2k}{\text{Exp}}_{k}+\beta_{1k}{\text{Asst}}_{ijk}\text{:}{\text{Exp}}_{k}+U_{k}+W_{jk}$$

Where the grand mean ($\beta_{0}$) represents the intercept; the coefficient $\beta_{1}$ denotes the fixed-effect logistic regression coefficient associated with Assistance; $\beta_{2k}$ represents the fixed-effect coefficients for the different Experience; the terms $\beta_{1k}$ and $\beta_{2k}$ capture the fixed-effect coefficients for the interactions between Assistance and the Experience; the random intercepts *U*_*k*_ and *W*_*jk*_ were assumed to be independent across Experience (*k*) and images (*j*) within the same levels of experience (*k*); the variable $i=1,\ldots ,I_{j}$ denotes the Level 1 indicator for individual participants (*i*); $j=1,\ldots ,J_{k}$ denotes the Level 2 indicator for images (*j*); and k=1,…,K denotes the Level 3 indicator Experience (*k*), where $K=4,J_{1}=5,J_{2}=2,J_{3}=2,\text{and}\;J_{4}=2$. Model 1 (the full model) includes interaction terms between Experience and Assistance, while Model 2 excludes Assistance as a fixed effect. Note that the variable for assistance status is Asst, and experience levels are Exp.

##### [H1b] The participants’ answers are more similar to the assisting tool suggestion when assisted than the unassisted one.

We evaluate this hypothesis to ensure that the assisting tool affects the participants’ decision when diagnosing the test images with the AISIM strategy. We used a mixed-effect logistic regression model to evaluate the hypothesis. The outcome variables used in this study are diagnostic similarities. The similarity variable takes a value of 1 if the participants’ answers are similar between AI suggestions and 0 if not similar between AI suggestions. We considered the effects of diagnostic status. The effects variables considered are assistance status (Assistance: takes the value of 1 if assisted and 0 if unassisted) and levels of experience (Experience :0 if general practitioners, 1 if residence radiologist, 2 if non-hepatobiliary radiologists, and 3 if hepatobiliary radiologists).

Similar to investigating the effect of the assisting tool on diagnostic status, two-level mixed-effects logistic regression models (Models 3–4) were performed to investigate the effect of the AI assisting tool on the similarity between AI’s answers and the participants’ answers. Assistance was a fixed effect, while individual participants (*i*), images (*j*), and Experience (*k*) were random effects. The models assumed that observations ($y_{111},\ldots ,y_{ijk}$) are independent of answers similarity or non-similarity result, and $Y_{ijk}∼\text{Bernoulli}\;(\pi_{ijk})$, where $\pi_{ijk}$ are probabilities of answers similarity to the assisting tool suggestion for individual participants (*i*), Images (*j*), and Experience (*k*), based on the dependent variable as a diagnostic status hence:


$$\text{Logit}(\pi |{\text{Asst}}_{i},{\text{Exp}}_{k},U_{k},W_{jk})=\beta_{0}+\beta_{1}{\text{Asst}}_{ijk}+\beta_{2k}{\text{Exp}}_{k}+\beta_{1k}{\text{Asst}}_{ijk}\text{:}{\text{Exp}}_{k}+U_{k}+W_{jk}$$

Where the grand mean ($\beta_{0}$) is the intercept term; the $\beta_{1}$ is the fixed-effects logistic regression coefficient corresponding to the assisted and unassisted statuses; $\beta_{2k}$ are the fixed-effect coefficients at the levels of experience; the $\beta_{1k}$ and $\beta_{2k}$ are the fixed-effect coefficients for interactions between the assistance groups for the experience levels; the random intercepts *U*_*k*_ and *W*_*jk*_ were assumed to be independent across Experience (*k*) and images (*j*) within the same Experience (*k*); the $i=1,\ldots ,I_{j}$ is the level 1 indicator for the individual participants (*i*); $j=1,\ldots ,J_{k}$ is the level 2 indicator for the images (*j*); and k=1,…,K is the level 3 indicator for the Experience (*k*) ($K=4,J_{1}=5,J_{2}=2,J_{3}=2,J_{4}=2$). Model 3 (the full model) includes interaction terms between experience level and Assistance. Finally, Model 4 excluded Assistance as a fixed component.

#### [Q2] How did different User Interface (UI) elements of the assisting tool impact the users’ decisions?

##### [H2’s] Elements in the user interface: AI’s confidence, AI’s viewing angle, and AI’s attention area impacts on participants’ decisions.

To test these hypotheses, we use the AISIM strategy by constructing a multiple logistic regression model in which AISIM was the dependent variable and AI confidence (high AI confidence (>50%) and low AI confidence (≤50%)), AI suggestion for the viewing angle (correct prediction and incorrect prediction), and the AI attention area were fixed effects (correct AI attention area (IoU between ground truth ROI and AI attention > 80), undecided AI attention (IoU between ground truth ROI and AI attention 20≥ and ≤80), and incorrect AI attention area (IoU between ground truth ROI and AI attention < 20)). Three independent variables were derived from participants’ final diagnoses after reviewing the image during the experiment. These were considered factors influencing the AISIM status of radiologists: AI’s confidence (Confidence: coded as 0 for low confidence and 1 for high confidence), AI’s attention area (Attention: coded as 0 for incorrect, 1 for undecided, and 2 for correct), and AI’s viewing angle (View; coded as 0 for incorrect and 1 for correct). The associations between AISIM status and AI’s confidence, AI’s attention area, and AI’s viewing angle (Model 5) were measured using multivariate binary logistic regression models [45], and the low confidence, incorrect AI’s attention area, and AI’s viewing angle incorrect were used as the reference group for the model, respectively.

Multivariate binary logistic regression is a statistical model used to assess the effect of several predictor factors when we have a dichotomous outcome. In this study, let $Y_{i}=(Y_{i1},Y_{i2},\ldots ,Y_{in})$ denotes the binary response of AISIM status (1 if the participants’ answers are similar between AI suggestions, and 0 if they are not similar between AI suggestions) for the *ith* subject. We note that the response *Y*_*ij*_ is recorded for each subject i,i=1,…,N at time j,j=1,…,n. This study collected all measurements from *N* = 11 subjects, with each subject reviewing *n* = 150 images. Model 5 assumes $Y_{i}∼\text{Bernoulli}\;(\pi_{ij})$. Our multivariate binary logistic regression model would have the form


$$\log\left(\frac{\pi_{ij}}{1-\pi_{ij}}\right)=\beta_{0}+\beta_{1}{\text{Confidence}}_{i}+\beta_{2}{\text{Attention}}_{i}+\beta_{3}{\text{View}}_{i}+\alpha_{i}$$

Where $\pi_{ij}$ represents the probability of a positive AISIM status in Model 5; $\beta_{0}$ is the intercept; $\beta_{1}$, $\beta_{2}$, and $\beta_{3}$ are the logistic regression coefficients for AI’s confidence, attention area, and viewing angle, respectively; the random effect $\alpha_{i}$ is assumed to follow a normal distribution with mean zero and constant variance, $\alpha_{i}∼N(0,\sigma_{\alpha }^{2})$ [38]. Note that the variable for AI’s confidence is denoted as Confidence, the AI’s attention area is Attention, and the AI’s viewing angle is View.

### User experiments

This research utilized retrospective data from the BiTNet experiment^1^. The participants consisted of 11 doctors with four different levels of experience in diagnosing abnormalities in ultrasound images of the human biliary tract. All participants have been working at Srinagarind Hospital, which is part of the Faculty of Medicine at Khon Kaen University in Khon Kaen, Thailand. Annually, the Department of Radiology of Srinagarind Hospital deals with an estimated 50,000 cases of the hepatobiliary risk group.

To minimize the learning effect during the user study, the task order was randomized from one participant to another. The participants were divided into 2 groups: Unassist  = > Assist and Assist  = > Unassist. Group 1 (Unassist  = > Assist) participants diagnosed the test images with the unassisted condition first. Then, they were asked to diagnose the test images with the presence of the assisting tool. Group 2 (Assist  = > Unassist) participants started with the assisting tool first, followed by the unassisted condition. There were washout periods of 4 weeks between the two testing sessions. The descriptions of the number of participants in each group are shown in Table 1. Additionally, the participants did not have access to other information related to the subject in the ultrasound images, such as gender, age, and symptoms while diagnosing the test images.

**Table 1:** The number of participants analyzing ultrasound images was divided into two groups. The first group (Group 1) did not use the assisting tool initially and then employed the assisting tool after a one-month washing period. The second group (Group 2) followed the reverse order, using the assisting tools first and then switching to not using the assisting tool after the same one-month washing period.

Group of doctors	Years of experience	Number of group 1	Number of group 2
General Practitioners (GP’s)	0	2	3
Residence Radiologists	1 to 5	1	1
Non-hepatobiliary radiologists (Non-HB Radiologists)	6 to 10	1	1
Hepatobiliary radiologists (HB Radiologists)	> 10	1	1

For each experiment, 50 ultrasound images were randomly rearranged twice. The first one was employed as a testing with AI assisting condition, whereas the second one was used as an experiment without AI assisting. The complete set of images is collected from all possible viewing angles and comprises 43 images from 14 abnormal classes and 107 images from the normal class. The ground truth of the 150 ultrasound images was confirmed by more precise methods, e.g. CT MRI, and biopsy.

**Fig 3:**
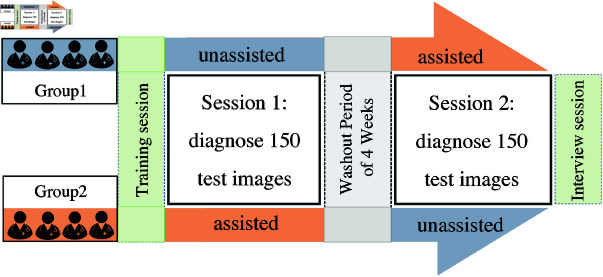
The experiment was designed to evaluate the participants’s performance when assisted vs unassisted. Group 1 diagnosed 150 test images without the assisting tool in the first session, then diagnosed the same test set with the assisting tool in the first session. Group 2 diagnosed 150 test images with an assisting tool in the first session, and then diagnosed the same test set without the assisting tool in the second session.

Assisted participants were asked to diagnose the image as either one of the 14 abnormality classes or a normal class while observing the assisting tool. The user interface of the assisting tool is composed of five parts: viewing angle prediction, AI suggestion, AI attention area, AI confidence, and top 3 suggestions. The user interface of the assisting tool is shown in Fig 3

### User interview questions

After the user experiments, we hold an interview section for every participant. The questionnaire is open-ended, and the answers are analyzed by grouping similar answers and counting the number of occurrences. The topics of the interviews are as follows,

- Did the assisting tool improve your confidence when the AI suggestion was the same as your answer? How about when the suggestion was against your decision?
- How often do you consider AI suggestions?
- Once you have decided, do you still need the assisting tool?
- Which user interface elements impact your decisions the most?
- Suggestions and more feedback on the assisting tool.

## Results and discussion

### [A1] Assisting improved diagnosis performance of the participant across all levels of experience.

We started by testing Hypothesis *H1a* (The participants have higher accuracy when assisted with the assisting tool than when unassisted). Table 2 shows that the *p*-value of the assisted condition was less than 10^−15^, which means the assisted condition had an impact on the diagnostic accuracy, and when the participants were using the assisting tool, they had about 3.326 times the odds of **making a correct final diagnosis**, compared with the same participants reviewing the image without the assistance (OR = 3.326, *p*-value <10^−15^). Further analysis shows that experience levels also greatly impacted the accuracy (*p*-value <0.05). The odd ratios of the levels of experience showed that Non-HB radiologists (5.667 times), resident radiologists (4.417 times), and HB radiologists (1.756 times) have higher odds of making correct diagnoses than general practitioners.

**Table 2:** Impacts of the assisting tool and participants’ experience levels on the accuracy of diagnosis. (‘***’ $p\leq 10^{-15}$, ‘**’ p≤0.01, ‘*’ p≤0.05, and reference category).

Predictor	Diagnostic Accuracy		
	Odd Ratio	95% CI	*p*-value
Assisted	3.326	(2.741, .871)	2.2*e*–16***
Levels of experience			0.02853*
Resident Radiologists vs GP’s	4.419	(1.539, 12.689)	
Non-HB Radiologists vs GP’s	5.577	(1.940, 16.033)	
HB Radiologists vs GP’s	1.740	(0.610, 4.966)	

Additionally, we found that sessions of the experiments impacted the accuracy, and the second session had higher average accuracy than the first session, 68.242±19.773% and 72.242±16.241%, respectively. After we extracted information from the interview session, we found that although the washout period was enough to allow participants to forget about their decision, the participants still remembered the proportion of the normal cases and the abnormal cases, which normal cases had a higher proportion than the abnormal cases.

**Fig 4:**
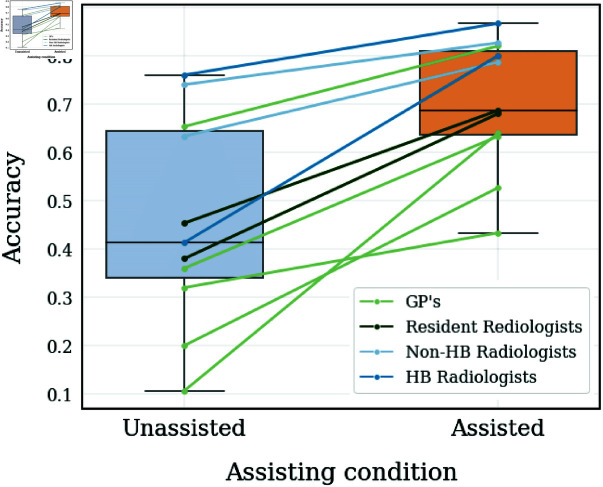
Comparison between accuracies of participants from different levels of experience when assisted and unassisted.

To confirm that the assisting tool plays an important role in the diagnosis accuracy, we evaluated Hypothesis *H1b* (The participants’ answers are more similar to the assisting tool suggestion when assisted than the unassisted attempt). Table 3 shows that the *p*-value of the assisted condition was less than 10^−15^, which means the assisted condition had an impact on the diagnostic similarity, and the decisions of the participants had about 4.753 times odd of **following the AI suggestion when they used the assisting tool**, compared to not using the tool (OR = 4.753, *p*-value <10^−15^). From Table 2 and Table 3, we can conclude that the participants tended to follow the assisting tool suggestion, which improved their diagnosis accuracy across all experience levels. Figure 4 demonstrates improvement among all participants when assisted with BiTNet.

**Table 3:** Impact of the assisting tool and participants’ experience levels on AISIM. (‘*’ $p\leq 10^{-15}$ and reference category).

Predictor	Diagnostic AISIM		
	Odd Ratio	95% CI	*p*-value
Assisted	4.753	(3.929, 5.748)	2.2*e*–16*
Levels of experience			0.040
Resident Radiologists vs GP’s	3.623	(1.296, 10.133)	
Non-HB Radiologists vs GP’s	5.064	(1.806, 14.196)	
HB Radiologists vs GP’s	1.584	(0.572, 4.385)	

#### The impact of the AI assisting tool on AISIM when the AI was wrong

We evaluate the impact of the assisting tool on the user’s decision, see if the assisting tool influences the users’ decision, when the AI made incorrect suggestions: when all top three AI suggestions were incorrect, and when the top AI suggestion was incorrect.

**[a] The top three AI suggestions were incorrect**

We assessed the impact of the AI assisting tool on the AISIM in cases where the top three AI predictions were incorrect. As shown in Table 4, the *p*-value for the assisted condition was less than 10^−3^, indicating a significant impact of the AI assisting tool on diagnostic similarity. Participants had about 3.779 times the odds of following the AI suggestion, even when all top-3 AI predictions were incorrect, compared to not using the tool (OR = 3.779, *p*-value <10^−15^).

**Table 4:** Impact of the assisting tool and participants’ experience levels on AISIM when the top-3 AI suggestions were incorrect. (‘*’ $p\leq 10^{-3}$ and reference category).

Predictor	Diagnostic AISIM		
	Odd Ratio	95% CI	*p*-value
Assisted	3.779	(1.631, 8.753)*	1.2*e*–4*
Levels of experience			0.509
Resident Radiologists vs GP’s	1.376	(0.457, 4.142)	
Non-HB Radiologists vs GP’s	0.532	(0.166, 1.699)	
HB Radiologists vs GP’s	0.684	(0.220, 2.126)	

**[b] The top AI prediction was incorrect**

We also evaluated the cases when only the top AI prediction was incorrect. Table 5 reveals that the *p*-value for the assisted condition was less than 10^−8^, demonstrating a substantial effect on diagnostic similarity. Participants had about 3.875 times odd of following the AI suggestion when using the assisting tool, despite the AI’s incorrect top prediction, compared to not using the tool (OR = 3.875, *p*-value <10^−8^).

**Table 5:** Impact of the assisting tool and participants’ experience levels on AISIM when the top AI suggestion was incorrect. (‘*’ $p\leq 10^{-8}$ and reference category).

Predictor	Diagnostic AISIM		
	Odd Ratio	95% CI	*p*-value
Assisted	3.875	(2.406, 6.243)*	7.4*e*–9*
Levels of experience			0.179
Resident Radiologists vs GP’s	1.072	(0.586, 1.960)	
Non-HB Radiologists vs GP’s	0.691	(0.367, 1.299)	
HB Radiologists vs GP’s	0.496	(0.256, 0.962)	

These findings indicate that even when the assisting tool provided incorrect answers, participants aligned their decisions with the tool’s suggestions. Furthermore, these effects were independent of the radiologists’ experience levels, meaning that regardless of level of experience, participants were higher odds time to following the AI’s suggestion.

### [A2] AI confidence has the highest impact among other user interface elements on participants’ decisions.

To understand how user interface elements of BiTNet impact the participant’s decision, we analyzed relations of the AISIM metric, the similarity between participants’ final answers and the AI suggestions, and the expression of different UI elements.

From Table 6, the *p*-values of AI confidence, AI suggestion for the viewing angle, and AI attention area indicate that all three UI elements impacted the user decision. In addition, AI confidence had a significantly higher impact on the metric, *p*-values <10^−15^, compared to the others, *p*-values <10^−2^ for both AI suggestion for the viewing angle and AI attention area.

**Table 6:** Impact of user interface elements: AI’s confidence, AI’s attention area, and AI’s viewing angle on AISIM, the similarity between participants’ answers and the AI suggestions. (‘***’ $p\leq 10^{-15}$, ‘**’ p≤0.01, and reference category)

Predictor	Diagnostic *AISIM*		
	Odd Ratio	95% CI	*p*-value
**AI confidence**			2*e*–16***
high vs low	2.927	(2.266 , 3.781)*	
**AI attention**			1.135e-3**
undecided vs incorrect	1.449	(1.021 , 2.056)*	
correct vs incorrect	12.978	(2.910 , 3.781)*	
**Viewing angle prediction**			9.217e-3**
correct vs incorrect	1.350	(1.077 , 1.693)*	

We then considered the Odd Ratio (OR) of each user interface element. For AI confidence, we found that when the participants saw AI prediction with high confidence (confidence >50%), they had about 2.927 times the odds of following the AI abnormality prediction compared to when the participants saw AI prediction with low confidence (confidence ≤50%). Furthermore, the participants had 12.978 times the odds of following the AI abnormality prediction when they noticed correct AI attention areas (IoU >0.80) than when they found the incorrect attention areas. IoU was calculated by the ratio between the intersection of the ground truth ROI area and the AI attention area and the union of the ground truth ROI area and the AI attention area. Finally, when considering the odd ratio of the AI suggestion for the viewing angle, we found that participants have only 1.35 times the odds of following AI abnormality prediction when the viewing angle prediction is correct compared to when the viewing angle prediction is incorrect. All in all, participants tend to follow the AI abnormality prediction when the user interface shows higher AI confidence, the correct AI attention area, and the correct AI viewing angle suggestion.

### User interview results

We conducted an user interview regarding the use of the assisting tool to diagnose upper abdominal diseases through an ultrasound image, focusing on users’ experiences, opinions, and feelings. After the user study were completed, the interviews were performed by a senior researcher and the section being audio-recorded. The interview results were then transcribed and analyzed. The result are shown in Tables 7 and 8. From an overall view, interview answers are summarized as follows,

- The participants felt more confident with their final decision when the assisting tool suggested the same answer to their diagnoses.
- When the suggestions differed, they carefully investigated the ultrasonography image and the UI elements again before making a final decision.
- Participants used the assisting tool with almost all test images to compare their diagnosis with the AI and to support when the images were ambiguous to diagnose.
- Most participants always considered the assisting tool’s suggestion after making their decisions.
- Some of the participants noticed that there were more images from normal cases than images from abnormal cases, so they confirmed their decisions with the assisting tool.
- 36% of the participants considered the AI attention area, followed by the AI confidence. (impacts of AI elements)
- 18% of the participants considered the AI confidence followed by the AI attention area. (impacts of AI elements)
- Participants could not remember the decisions they made in the first session, but they remembered that the proportion of the normal cases was higher than the abnormal cases. (washout period is effective)
- The top suggestion for the assisting tool improvement was that the tool should allow the user to investigate more than one image per case. It should also include patient information for the diagnosis.
- Opinions about the assisting tool were that AI increased their confidence because it helped when the images were ambiguous, the AI acted as a second opinion, and the assisting tool helped when both their decisions and the AI suggestion were normal cases.

**Table 7:** Initial coding framework with frequency counts from the interview results from questions Q1-Q5

		#	%
**Q1**	**What is the interviewees’ opinion on the assisting tool?**		
	Couldn’t recognize the image.	8	73
	Were not asked.	3	27
**Q2**	**Did the interviewees check every image with the assisting tool, and when did they use it?**		
	**(For example, only looking when- they thought it was abnormal)**		
	Looked at the AI attention first, then the AI confidence.	4	36
	Looked at the AI confidence first, then the AI attention.	2	18
	Only checked the top-3 AI results.	1	9
	Checked the AI confidence first, then the AI attention, followed- by the top-3 AI suggestions.	2	18
	Viewed the Viewing Angle first, then the top-3 AI suggestions, followed by AI attention.	1	9
	Looked at the AI attention first, followed by the top-3 AI suggestions, then the Viewing Angle.	1	9
**Q3**	**What aspects of the assisting tool did interviewees find helpful- in their decision-making, and what were the key factors- ranked by importance?**		
	**Viewed only certain images**	**9**	**82**
	- Mainly looked at abnormal cases when the ultrasound images were unclear.	2	18
	- To compare diagnostic results.	4	36
	- Mostly in normal cases when the ultrasound images were unclear.	3	27
	**Viewed all images**	**2**	**18**
	- Mainly looked at abnormal cases.	1	9
	- Focused primarily on the AI attention.	1	9
**Q4**	**Did using the assisting tool increase confidence when- predictions matched, and what did they do when predictions- didn’t match?**		
	**When predictions matched**		
	For images where they weren’t confident, using AI increased confidence.	3	37
	It helped increase confidence in certain images, mainly through the AI attention.	1	9
	Increased confidence when predictions matched.	7	64
	**When predictions did not match**		
	Trusted their own judgment (mostly).	4	36
	Reviewed the images again.	7	64
**Q5**	**Did interviewees think they would continue using the Assist- tool if they decided an image was normal or abnormal?**		
	Reviewed some ultrasound images, especially in normal cases.	2	18
	Felt more confident overall.	8	73
	Reviewed some ultrasound images, especially in abnormal cases.	1	9

**Table 8:** Initial coding framework with frequency counts from the interview results from questions Q6 and Q7.

		#	%
**Q6**	**Suggestions for Improving the assisting tool or the- Experimental Process**		
	The top-3 AI suggestions should not be included as they- increase hesitation for doctors.	1	6
	Multiple angles of the images should be considered.	5	28
	AI cannot differentiate artifacts, which might affect- decision-making.	1	6
	the AI attention is still inaccurate, leading to incorrect- AI predictions.	1	6
	The assisting tool has sufficient functions; more- would increase the doctor’s workload.	6	33
	Most often, patient information is used in- conjunction with the diagnosis.	2	11
	Some ultrasound images have markers that may- cause confusion.	1	6
	Abbreviations for abnormality names should be- clearly stated.	1	6
**Q7**	**Overall Opinions on the assisting tool**		
	**AI helps increase confidence**	**9**	**82**
	- It helps weigh options before making a decision and - AI feels like a supportive partner.	4	36
	- It is useful in ambiguous cases.	2	18
	- AI is somewhat helpful to a certain extent.	2	18
	- It’s helpful when AI matches in normal cases.	1	9
	**Not very confident in AI**	**2**	**18**
	- In some images where the interviewee is already- confident, they don’t refer to AI, especially in- abnormal cases like stone polyp.	1	9
	- In some images where it’s clear that AI is incorrect, they choose not to trust AI.	1	9
	**Provided additional recommendations**		
	- Normally, multiple angles are checked.	3	25
	- Having AI is still better than not having it.	1	8
	- Sometimes they consider an abnormal case that’s- not among the choices.	1	8
	- The assisting tool takes time in decision-making.	2	17
	- Often consider patient information alongside AI results.	2	17
	- Most often, patient information is used in conjunction- with the diagnosis.	1	8
	- Some images with markers may cause confusion.	1	8
	- Sometimes they choose answers outside of the top-3 suggestions.	1	8

We conducted a thematic analysis to obtain better-organized insights and found two themes.

**Theme 1 Confidence in AI**

The theme “Confidence in AI” reveals how the assisting tool affected the trust and confidence of the interviewees in their diagnostic decisions. Many participants indicated that the tool boosted their confidence, especially when the AI’s predictions aligned with their judgments. One participant expressed this sentiment by saying, “Confidence increased when the predictions matched.” It suggested that the assisting tool was particularly effective when it confirmed the doctors’ own diagnostic assessments, reinforcing their decisions and providing additional assurance in uncertain cases. However, not all respondents fully relied on AI, especially when the predictions diverged from their own evaluations. In such cases, some doctors choose to rely more on their own judgment. As one interviewee stated, “I trusted my own judgment when the predictions didn’t match.” This highlights a limitation of AI assisting tools in clinical settings While AI can augment decision-making, human expertise remains the primary guide, particularly when there is a discrepancy between AI’s suggestions and doctors’ decisions. Furthermore, some respondents noted that AI was helpful in certain images or cases where they felt less confident. In these situations, the tool acted as a form of reassurance. For instance, one respondent mentioned, “For images where I wasn’t confident, using AI increased my confidence.” This indicates that the tool served as a valuable secondary opinion in cases where doctors faced uncertainty, especially in diagnosing complex or ambiguous images. Despite the increase in confidence provided by the tool, some participants acknowledged that they would not rely solely on AI but rather use it as a supplementary tool. One participant remarked, “AI is helpful to a certain extent, but I still rely on my own judgment.” This reflects the nuanced relationship between AI and human decision-making, where AI is viewed as a companion or support rather than a replacement for clinical expertise. In summary, the theme of Confidence in AI shows that while the assisting tool can enhance doctors’ confidence in their diagnoses, particularly when AI predictions align with their own, it does not replace the critical role of human judgment. The tool is perceived as a helpful assistant, but its effectiveness depends heavily on the accuracy of its predictions and its ability to provide reassurance without causing doubt or hesitation when discrepancies arise.

**Theme 2 Suggestions for Improvement**

The theme “Suggestions for Improvement” highlights the feedback from interviewees aimed at enhancing the accuracy and efficiency of the assisting tool. Many respondents identified specific issues they encountered during use, suggesting that addressing these could significantly improve the tool’s ability to support clinical decision-making. One of the most common suggestions was the need for AI to consider multiple angles of the ultrasound images more effectively. As one participant noted, “The AI should consider multiple angles of the image because it might miss certain dimensions at times.” This emphasizes the importance of comprehensive image analysis for more accurate diagnoses. Additionally, some respondents pointed out that the AI struggled to differentiate between relevant features and artifacts, which can complicate the decision-making process. One interviewee mentioned, “The AI couldn’t separate artifacts well enough, which made it difficult to make decisions.” This is a significant limitation that, if addressed, could enhance the AI’s diagnostic precision. Another key suggestion was to reduce the display of the AI’s Top-3 AI suggestions, as this sometimes caused hesitation among physicians when making decisions. One respondent said, "There shouldn’t be the top-3 AI suggestions because it made doctors more hesitant to decide." While the top-3 suggestion can be helpful in some cases, it might also create confusion, making it harder for physicians to rely on their own judgment. Several respondents also called for improvements to the AI attention area, which was often described as inaccurate. One interviewee explained, "The AI attention is still not accurate enough, leading to incorrect predictions." This underscores the need to refine this UI element so that the AI can pinpoint abnormal areas more precisely and reliably. Despite the various suggestions, some respondents felt that the assisting tool already had good functionality, warning that adding too many UI elements might increase the doctors’ workload unnecessarily. One participant remarked, “The tool has good functionality already; adding more might make doctors work harder.” This feedback suggests that while improvements are needed, developers should balance new functionalities with the potential impact on clinical workflows. Overall, these suggestions reflect the desire to make the assisting tool more accurate and user-friendly, while ensuring that it integrates smoothly into real-world medical settings. The focus on enhancing both the diagnostic accuracy and the usability of the assisting tool highlights the importance of creating a system that not only supports but simplifies decision-making for physicians.

### Ethical considerations and limitations

While our results demonstrate the utility of AI assistance in medical imaging diagnosis, several important ethical considerations must be addressed in future work. First, ensuring patient privacy and data security when implementing such systems at scale remains crucial [46]. Second, regular auditing for potential biases in AI suggestions across different patient demographics is essential for maintaining fairness in healthcare delivery [47]. Third, clear protocols must be established for documenting how AI suggestions influence clinical decisions to maintain transparency and accountability [48]. Our study was conducted in a controlled research environment, and future work should investigate how these findings translate to real-world clinical settings where additional factors like time pressure, varying image quality, and diverse patient populations may impact the tool’s effectiveness. Furthermore, longitudinal studies are needed to assess how sustained use of AI assistance affects clinical decision-making patterns and doctor-patient relationships over time.

## Conclusion

In this paper, we proposed AISIM strategy to measure the impact of an AI when used as an intelligent assisting tool. The tool we focused on in this research is BiTNet, an assisting tool for diagnosing abnormalities in the human biliary tract via ultrasound image. We analyzed BiTNet user interface through the strategy with user experiments on 11 healthcare practitioners with mixed and fixed effect logistic regressions.

We proposed to measure the impacts of the tools on user decisions via the AISIM, and the similarity between the user diagnoses and the AI suggestions. In analysis, the AISIM is set as the dependent variable, and the elements, which aimed to measure the impacts, are set as fixed effects.

In our user experiment, the main findings from our proposed analysis demonstrate that the assisting tool impacts the participants’ performance, and the tool improved the accuracy of the participants across different levels of experience. Furthermore, the user experiment showed that AI confidence had the highest impact on participants’ decisions, where they tend to follow AI abnormality prediction when AI confidence is high (OR = 2.927, *p*-value<10^−15^). They were followed by the AI attention area, in which participants had 12.978 times the odds of following AI abnormality prediction when they saw the correct AI attention area (OR = 12.978, *p*-value<10^−2^). The interview also shows that the assisting tool increased participants’ confidence in different situations. The participants felt more confident with their final decision when the assisting tool suggested the same answer to their diagnoses. When the suggestions were different, they carefully investigated the ultrasound image and the user interface elements again before their final decision. Moreover, both the statistical analysis and the interview agreed that the AI confidence and the AI attention area impacted participants’ decisions. However, the statistical analysis illustrated that AI confidence is the UI element that has the highest impact on the participants’ decisions, followed by the AI attention areas, while the user interviews indicated that the AI attention area was the first UI element considered by most participants then the AI confidence. Although the results of the user experiment are beneficial for measuring the impacts of the User Interface, we are looking forward to evaluating more AI tools with AISIM to investigate more on the metric. Moreover, more rigorous data collection methods such as eye tracking and interaction log collection can also be used in subsequent studies to extract more hidden insight and strengthen the studies.

## Footnotes

^1^ The study was conducted according to the guidelines of the Declaration of Helsinki and approved by the Human Ethics Committee of Khon Kaen University, Khon Kaen, Thailand, based on the ethics of human specimen experimentation of the National Research Council of Thailand (HE631353; approved date June 30, 2020. The recruitment period of the study was between August 1, 2020 and March 19, 2021. All participants were asked to sign the consent forms before participating. The experiment data were fully anonymized before accessing on June 15, 2021.
